# Postmortem Findings in Patient with Guillain-Barré Syndrome and Zika Virus Infection

**DOI:** 10.3201/eid2401.171331

**Published:** 2018-01

**Authors:** Emilio Dirlikov, José V. Torres, Roosecelis Brasil Martines, Sarah Reagan-Steiner, George Venero Pérez, Aidsa Rivera, Chelsea Major, Desiree Matos, Jorge Muñoz-Jordan, Wun-Ju Shieh, Sherif R. Zaki, Tyler M. Sharp

**Affiliations:** Centers for Disease Control and Prevention, Atlanta, Georgia, USA (E. Dirlikov, R.B. Martines, S. Reagan-Steiner, C. Major, W.-J. Shieh, S.R. Zaki);; Puerto Rico Institute of Forensic Sciences, San Juan, Puerto Rico, USA (J.V. Torres);; Hermanos Meléndez Hospital, Bayamón, Puerto Rico, USA (G.V. Pérez);; Centers for Disease Control and Prevention, San Juan (A. Rivera, C. Major, D. Matos, J. Muñoz-Jordan, T.M. Sharp);; US Public Health Service Commissioned Corps, Rockville, Maryland, USA (T.M. Sharp)

**Keywords:** Guillain-Barré syndrome, Zika virus, vector-borne infections, postmortem investigation, infectious disease pathophysiology, Puerto Rico, viruses

## Abstract

Postmortem examination results of a patient with Guillain-Barré syndrome and confirmed Zika virus infection revealed demyelination of the sciatic and cranial IV nerves, providing evidence of the acute demyelinating inflammatory polyneuropathy Guillain-Barré syndrome variant. Lack of evidence of Zika virus in nervous tissue suggests that pathophysiology was antibody mediated without neurotropism.

Guillain-Barré syndrome (GBS) is an uncommon autoimmune disorder characterized by progressive, bilateral weakness and diminished deep tendon reflexes due to peripheral nerve damage. GBS is typically triggered by an acute infection and, less frequently, by vaccination (*1*). GBS has been associated with infection by Zika virus, a flavivirus transmitted primarily by *Aedes* species mosquitoes (*2*), and countries have reported increased GBS incidence during Zika virus outbreaks (*3*–*5*). Reports suggest Zika virus may result in a hyperacute immune response or have a direct viral neuropathic effect contributing to GBS (*6*). Further, case reports and series have noted higher rates of cranial neuropathy, such as facial palsy and paresthesia, among GBS patients with evidence of Zika virus infection, suggesting that the cranial nerves may be targeted by either virus or antibody (*3*,*6*–*9*).

Mortality rates among GBS patients in North America and Europe vary from 3% to 7% (*1*), and death often results from respiratory failure, autonomic dysfunction, or deep vein thrombosis (*10*). In Puerto Rico, the GBS in-hospital mortality rate before the introduction of Zika virus was estimated at 4% (*11*). Postmortem investigations of GBS are rare, but results may indicate underlying pathophysiologic mechanisms.

During a Zika virus epidemic in Puerto Rico in February 2016, an islandwide surveillance system was implemented to identify GBS cases and provide Zika virus diagnostic testing (*9*). Fatal GBS cases could be reported, and postmortem investigations were incorporated into an established fatal case surveillance system (*12*). Such investigations were implemented to clarify the pathophysiology of GBS patients with Zika virus infection.

## The Study

**Figure 1:**
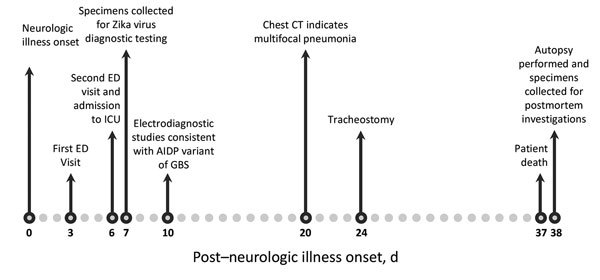
Timeline of key events surrounding the illness of a patient with GBS and confirmed Zika virus infection, Puerto Rico, 2016. AIDP, acute inflammatory demyelinating polyneuropathy; CT, computed tomography; ED, emergency department; GBS, Guillain-Barré syndrome; ICU, intensive care unit.

In August 2016, a 78-year-old man living in the San Juan metropolitan area with a medical history of hypertension (for which he was taking amlodipine), diabetes, asthma, and prostate cancer visited a hospital emergency department with a 4-day history of worsening paresthesia of the lower and upper extremities and progressive bilateral lower and upper extremity weakness (Figure 1). Computed tomography without contrast of the head found no acute intracranial or other abnormalities. The patient was given albuterol nebulizer treatment, ipratropium bromide, and ceftriaxone and discharged home on the same day. Three days later, the patient returned to the emergency department with worsened weakness. He was admitted to the intensive care unit with respiratory distress and was intubated. Hospital staff suspected GBS due to monophasic illness progression, symmetric weakness, and loss of deep tendon reflexes. A 5-day course of intravenous immunoglobulin (30 g/d) was initiated, and serum and urine specimens were collected for Zika virus diagnostic testing. On day 7 after onset of neurologic illness, the patient had acute kidney injury and hyperuricemia. Electrodiagnostic studies performed 10 days post-onset identified demyelinating polyneuropathy consistent with GBS (Technical Appendix). Given clinical features and electrophysiologic findings, the patient met level 2 of the Brighton Collaboration Criteria for GBS diagnostic certainty (*13*).

The patient was mildly responsive by day 15 and thereafter became unresponsive but hemodynamically stable. On day 16, pneumonia was diagnosed, and a course of vancomycin was started. Multidrug-resistant forms of *Acinetobacter baumanii* were found in sputum (collected on day 14) and fecal samples (collected on day 18), and multidrug-resistant *Enterobacter aerogenes* and *Klebsiella pneumoniae* were isolated from sputum (collected on day 27). Computed tomography of the chest performed on day 20 indicated multifocal pneumonia with parapneumonic effusions involving the entire right lung and part of the left lung. Following an episode of cardiac arrest on day 23, tracheostomy for chronic respiratory failure was performed on day 24. The patient died on day 37 due to cardiac arrest and respiratory failure complicated by bilateral pneumonia, sepsis, and acute renal failure.

An autopsy was performed, and external evaluation noted edema of the left hand, scrotum, penis, legs, and feet, as well as decubitus ulcers on the back and in the gluteal area. Internal examination showed bilateral pleural effusions, hepatic congestion, ascites, and cardiomegaly. Microscopic examination showed focal pneumonia, acute tubular necrosis, and prostate adenocarcinoma. Brain examination showed subacute watershed infarcts of the left cerebral hemisphere; occasional perivascular lymphomononuclear cell infiltrates were also observed.

We detected Zika virus RNA by reverse transcription PCR (RT-PCR) in urine and Zika virus and dengue virus IgM antibodies in serum (both collected on day 7). Additional laboratory testing of premortem specimens was negative for *Shigella*, *Salmonella*, *Yersinia*, enteropathic isolates, *Campylobacter jejuni*, *Legionella*, *Mycoplasma pneumoniae*, HIV, influenza A and B, cytomegalovirus, and Epstein-Barr virus. We performed Zika virus RT-PCR and immunohistochemical (IHC) assays on formalin-fixed paraffin-embedded autopsy tissue specimens. We detected neither RNA nor antigen for Zika virus in any autopsy specimens tested (Zika virus RT-PCR, cranial nerve VII, sciatic nerve, and spinal cord including cauda equina; IHC, brain, spinal cord, sciatic nerve, and multiple solid organs).

**Figure 2:**
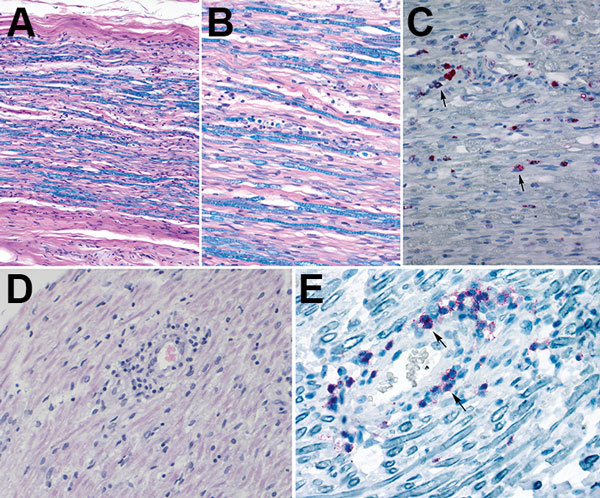
Histopathologic evaluation of tissue specimens collected postmortem from a patient with Guillain-Barré syndrome (acute demyelinating inflammatory polyneuropathy variant) and Zika virus infection, Puerto Rico, 2016. A, B) Luxol fast blue-periodic acid-Schiff myelin stain of sciatic nerves show patchy myelin loss and variable inflammation. Original magnification x10(A) and x20(B). C) Detection of CD68–positive cells (microphages) by immunohistochemistry (arrows) in sciatic nerve. Original magnification x20. D) Hematoxylin and eosin stain of cranial nerve IV shows perivascular lymphocytic infiltrate. Original magnification x20. E) Detection of T-lymphocytes by immunohistochemistry (arrows) in the same area where lymphocytic infiltrates were observed by hematoxylin and eosin stain. Original magnification x40.

We analyzed postmortem specimens of the peripheral and central nervous system and specimens from other organs by microscopic examination and special stains. A section of cranial nerve IV showed mononuclear lymphocytic infiltrate and mild myelin loss, while sections of the sciatic nerve showed inflammation-associated myelin loss. Myelin loss was highlighted by luxol fast blue staining (Figure 2). IHC staining for CD68 demonstrated an abundance of macrophages. Cranial nerves I, II, III, V, VI, and VII and spinal cord showed no substantial histopathologic findings. Pathology findings in lung, prostate, and spleen tissues were consistent with comorbid conditions. Findings for heart, testicle, adrenal gland, gastrointestinal system, and thyroid specimens were unremarkable.

## Conclusions

We report postmortem findings of a fatal GBS case (online Technical Appendix). Confirmation of Zika virus infection during acute neurologic illness and negative diagnostic test results for other potential GBS triggers (e.g., *C. jejuni*) provide strong evidence for Zika virus as the GBS trigger. Consistent with the patient’s electromyography results, histopathologic observation of demyelination of sciatic and cranial IV nerves provides phenotypic evidence of the acute demyelinating inflammatory polyneuropathy GBS variant. Although results from French Polynesia and Colombia found the acute motor axonal neuropathy variant (*3*,*9*), elsewhere, the acute demyelinating inflammatory polyneuropathy variant has predominated among GBS patients with evidence of Zika virus infection (*14*). Demyelination of both sciatic and cranial IV nerves is consistent with reported clinical signs of GBS patients with evidence of Zika virus infection. Despite reports of cranial neuropathy, including higher rates of facial palsy, ophthalmoplegia, and dysphagia, we observed no substantial pathologic findings in the patient’s cranial nerves I, II, III, V, VI, or VII. We found no evidence of direct infection of the peripheral or central nervous tissue, suggesting expected antibody-mediated pathophysiologic mechanisms for GBS were triggered by Zika virus and not neurotropism.

Our report has several limitations. First, findings from a single patient might not be representative. Second, because antecedent illness was denied, timing of Zika virus infection could not be defined; however, time-to-loss of detection of Zika virus RNA in the urine of patients with symptomatic Zika virus infection is a median of 8 days (*15*). Last, neurotropism concomitant to antibody-mediated damage cannot be ruled out because viral antigen and RNA may have been cleared before autopsy or false-negative laboratory results could have occurred. Further, Zika virus RT-PCR results were only available for cranial nerve VII, 3 sections of sciatic nerve, spinal cord, and cauda equina.

Despite these limitations, our findings provide crucial insights into GBS pathophysiology, specifically among GBS patients with evidence of Zika virus infection. Future investigations should collect postmortem specimens from GBS patients, particularly when death occurs soon after neurologic illness onset.

**Technical Appendix.** Results and recorded interpretation of electrodiagnostic studies performed 10 days post–neurologic illness onset in patient with Guillain-Barré syndrome (acute demyelinating inflammatory polyneuropathy variant) and Zika virus infection, Puerto Rico, 2016.
