# Supplementary material for: Postmortem Findings in Patient with Guillain-Barré Syndrome and Zika Virus Infection
Source: Emerg Infect Dis. 2018 Jan;24(1):114–7. doi: 10.3201/eid2401.171331 (PMC5749436; doi:10.3201/eid2401.171331)
Supplement: Supplementary file 1 — Technical Appendix. Results and recorded interpretation of electrodiagnostic studies performed 10 days post–neurologic illness onset in patient with Guillain-Barré syndrome (acute demyelinating inflammatory polyneuropathy variant) and Zika virus infection, Puerto Rico, 2016. [file 17-1331-Techapp-s1.pdf]

Article DOI: http://dx.doi.org/10.3201/eid2401.171331

Postmortem Findings in a Patient with Guillain-Barré Syndrome and Zika Virus Infection

Technical Appendix

The following are the available results and recorded interpretation of the electrodiagnostic studies performed on a patient with Guillain-Barré syndrome and Zika virus infection 10 days post–neurologic illness onset.

Technical Appendix Table 1. Results from electromyography studies of a patient with Guillain-Barré syndrome and Zika virus infection 10 days post–neurologic illness onset, Puerto Rico, 2016

					Spontaneous activity			
			Insertional activity		Fibrillation		Positive sharp wave	
Muscles	Roots*	Innervations	Right	Left	Right	Left	Right	Left
Upper extremities								
Deltoid	C5–C6	Axillary	Increased	—	+1	—	+1	—
Biceps	C5–C6	Musculocutaneous	None	—	0	—	0	—
First dorsal interosseous	C8–T1	Ulnar	None	—	0	—	0	—
Lower extremities								
Vastus medialis	L2–L4	Femoral	None	—	0	—	0	—
Tibialis anterior	L4–L5	Deep peroneal	Increased	—	0	—	+1	—
Gastrocnemius	S1–S2	Tibial	None	—	0	—	0	—

*C, cervical; L, lumbar; S, sacral; T, thoracic.

Technical Appendix Table 2. Results from nerve conduction studies of a patient with Guillain-Barré syndrome and Zika virus infection 10 days post–neurologic illness onset, Puerto Rico, 2016*

Nerves tested	Conduction velocity (m/sec)	Latency (ms)	Amplitude (mV)	Latency (ms)	Amplitude (mV)
Upper extremities					
Right median	36.1	19.7	0.008	4.7	3.0
Right ulnar	46.7	4.9	1.3	4.9	9.0
Lower extremities					
Right common peroneal	33.2	6.5	2.2	—	—
Right tibial	—	8.7	0.08	—	—
Right sural	—	—	—	3.8	2.7
Late responses†					
Right H reflex	—	Absent			
Right F wave	—	38.2			

*—, no test performed; absent, the test was performed, but no signal was found.
†Tests captured only conduction velocity and latency

Recorded interpretation: "Severely prolonged motor and sensory latencies, increased temporal dispersion, decreased velocities, absent H reflex, [and] prolonged F wave are highly suggestive of acute inflammatory demyelinating neuropathy as in Guillain-Barré syndrome."
